# Patient’s Perception of Outcome after Extracapsular Fractures of the Mandibular Condyle Differs from Objective Evaluation—Experience of a Third-Level Hospital

**DOI:** 10.3390/jcm13051395

**Published:** 2024-02-28

**Authors:** Michael Maurer, Tabea Klaes, Mathias Fiedler, Juergen Taxis, Johannes G. Schuderer, Waltraud Waiss, Maximilian Gottsauner, Johannes K. Meier, Torsten E. Reichert, Tobias Ettl

**Affiliations:** Department of Cranio- and Maxillofacial Surgery, Hospital of the University of Regensburg, Franz-Josef-Strauß-Allee 11, 93053 Regensburg, Germany

**Keywords:** mandibular condylar fracture, mouth opening reduction, malocclusion, facial nerve palsy, post-treatment pain, retromandibular transparotid approach, questionnaire

## Abstract

**Background**: The aim of this study is to assess patients’ subjective perception of treatment outcome after extracapsular fractures of the mandibular condyle. **Methods**: A questionnaire survey regarding facial nerve palsy (FNP), malocclusion, pain, reduction in maximum mouth opening (MMO) and further discomfort after 3, 6, and 12 months was carried out. Patients aged 18 or more presenting with an extracapsular condylar fracture between 2006 and 2020 were identified by purposive sampling Questionnaires were received from 115 patients. Fractures were classified on the basis of the pre-treatment imaging, the way of treatment was obtained from patients’ medical records. Data were analyzed using Pearsons’ chi-square-test, descriptive statistics and Student’s *t*-test. **Results**: 93.0% of the fractures were treated by open reduction and internal fixation (ORIF). MMO reduction was the most common post-treatment complication (55.6%). ORIF was associated with less pain after 3 months (*p* = 0.048) and lower VAS scores compared to conservative treatment (*p* = 0.039). Comminuted fractures were more frequently associated with post-treatment malocclusion (*p* = 0.048), FNP (*p* = 0.016) and MMO reduction (*p* = 0.001). Bilateral fractures were significantly accompanied by malocclusion (*p* = 0.029), MMO reduction (*p* = 0.038) and pain occurrence (*p* < 0.001). **Conclusions**: Patients report less pain after ORIF. Comminuted and bilateral fractures seem to be major risk factors for complications. Subjective perception of complications after extracapsular condylar fractures differs from objectively assessed data.

## 1. Introduction

Fractures of the mandibular condyle are reported to account for up to 45% of all mandibular fractures [1]. Despite the exceptional frequency of this kind of fracture, its management is discussed controversially in maxillofacial surgery [2,3]. Ellis identified malocclusion, mandibular hypomobility, deviation of the mandible, degenerative joint disease and iatrogenic injuries as major complications of this type of fracture [4]. Conservative treatment of condylar fractures is accomplished by using elastic bands, which can be usually deployed for fractures with no or minimal displacement, whereas surgical treatment is achieved by open reduction and osteosynthesis using miniplates or lag screws [5,6]. Potential complications after conservative treatment are mandibular deviation, malocclusion, ankylosis and internal derangement of the joint among others [7]. On the one hand, ORIF is reported to lead to better occlusal results, anatomic restoration and faster recovery rates than non-surgical techniques [8]. On the other hand, potential complications associated specifically with ORIF such as FNP and deranged occlusion cannot be neglected [9]. Some authors consider open and closed treatment to be equal in terms of MMO, mandibular deviation and restoring occlusion [10]. Thean et al. report a temporary malfunction of the facial nerve in 3.28% and a permanent nerve damage in 0.82% following ORIF. Moreover, they assessed a rate of post-treatment of malocclusion of 6.14% after conservative as well as open treatment [10]. Roozebom et al. refer temporary FNP in 12% of their cases, of which 95% achieved full recovery [11]. Lima et al. observed an incidence of 1.8% of FNP after the retromandibular approach [12]. In another study, 21% of the closed-treated patients and 2% of the open-treated patients showed post-treatment malocclusion [13]. In a recent study conducted in our own department, the post-treatment rate of malocclusion was 18.0%; temporary FNP occurred in 7.1% compared to 1.7% permanent FNP. MMO restriction was assessed in 13.5% of the cases [11]. Although various studies exist regarding the objective results of the treatment of extracapsular condylar fractures, there are a lack of data about subjective outcome and individual discomfort. Despite presumably high relevance, the incidence of chronic posttraumatic and postsurgical pain after fractures and fracture-related surgery is generally under-recognized [14]. In this context, our study is aimed at the assessment of postsurgical discomfort in terms of pain, FNP, malocclusion, and MMO reduction after surgical or conservative treatment of this certain kind of fracture. The study intends to provide information about the outcome from the patients’ perspective gathered by a questionnaire survey.

## 2. Materials and Methods

Inclusion criteria of the study were treatment of an uni- or bilateral extracapsular fracture of the mandibular condyle in our department of oral and maxillofacial surgery at Regensburg University Hospital, Germany, between 1 January 2006 and 31 December 2020 and age ≥ of 18 years or more at the date of the trauma. Intracapsular fractures were not included. Moreover, cases with extracapsular fracture on the one side and intracapsular fracture on the other side were not included to avoid confusion concerning the subjective outcome. Patients meeting this criteria were identified by purposive sampling. They were sent a printed postal questionnaire between 1 and 31 January 2022 so a minimum follow-up time of one year was granted. The questionnaire in German was developed exclusively for the present study (Table S1). Patients were requested to state their subjective feeling of occlusal disharmony, post-treatment pain, and reduction in maximum mouth opening (MMO) at three time measuring points: in short term (3 months), medium term (6 months) and long-term (12 months). Pain intensity was assessed according to a numerical rating scale (NRS) ranging from 0 to 10 (0 = no pain, 10 = worst pain). To assess FNP, the subjective perception of a restriction of the frontal, zygomatic, buccal or marginal mandibular branch, respectively, a visible disturbance of the innervated mimic muscles was retrieved. In the last section of the questionnaire patients were encouraged to verbalize further posttraumatic and postsurgical discomfort. The information acquired by the questionnaire survey were correlated to the patients` medical records. Comorbidities that affected the results could not be identified. Fracture localization (uni/bilateral), necessity for revision surgery, further fractures of the mandible, and polytraumatization were gathered from clinical documentation. By reference to preoperative imaging fractures were classified according to Spiessl and Schroll [15] (Table 1) and additionally the presence of comminution was assessed. Type VI fractures were not included in the study, as mentioned above. ORIF was accomplished by a retromandibular transparotid, preauricular, submandibular, or intraoral approach. Conservative treatment consisted of intermaxillary fixation by IMF screws (DePuy Synthes, Raynham, MA, USA) and elastics (Helago^®^, Heinz und Laufer OHG, Bonn, Germany). Osteosynthesis was carried out by 2 miniplates (Matrix Mandible Adaption Plate, thickness 1.0 mm combined with 6 mm screws, DePuy Synthes, Raynham, MA, USA). A 4-hole-plate was located along the posterior border of the mandibular ramus and a 3- or 4-hole-plate was placed along the anterior aspect of the condylar neck diverging to the first plate in antero-caudal direction. The double-plating technique is widely accepted and offers sufficient stability in a load-sharing concept [16,17]. Patients treated in other surgical ways were not included in the study. Malocclusion, FNP, MMO reduction and postoperative pain were correlated to the sex, method of treatment, Spiessl and Schroll classification, displacement, dislocation of the condylar head, comminution, fracture localization and concomitant mandibular fractures. FNP was furthermore correlated to neuromonitoring, revision surgery and approach. Data were analyzed by the use of SPSS 28.0. Data was analyzed using descriptive statistics, Pearsons’ chi-square-test and Student’s *t*-test. A *p*-value less than 0.05 was considered statistically significant.

## 3. Results

### 3.1. Base Data

Baseline data are shown in Table 2. The questionnaire was sent to 290 patients who underwent treatment of extracapsular fractures of the mandibular condyle at Regensburg University Hospital between 2006 and 2020 and fulfilled the inclusion criteria mentioned above. Replies were received from 115 patients (46 female (40.0%); 69 male (60.0%)). The response rate was 39.7%. The mean age was 41.89 ± 16.59 years (range 18–86 years).

**Figure 1:**
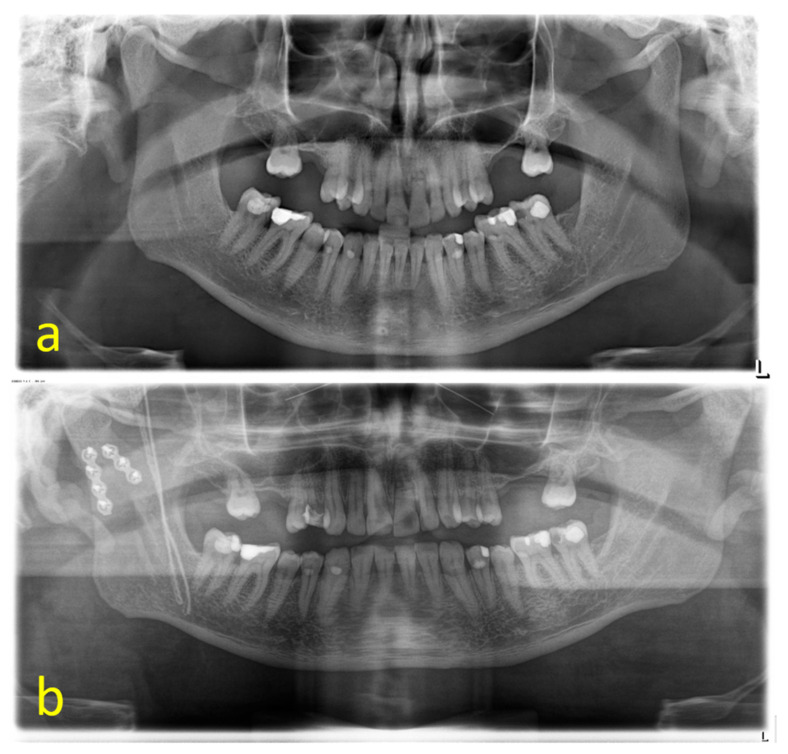
Type II fracture of the right mandibular condyle before (**a**) and after ORIF in double-plating technique (**b**) (L = left side).

**Figure 2:**
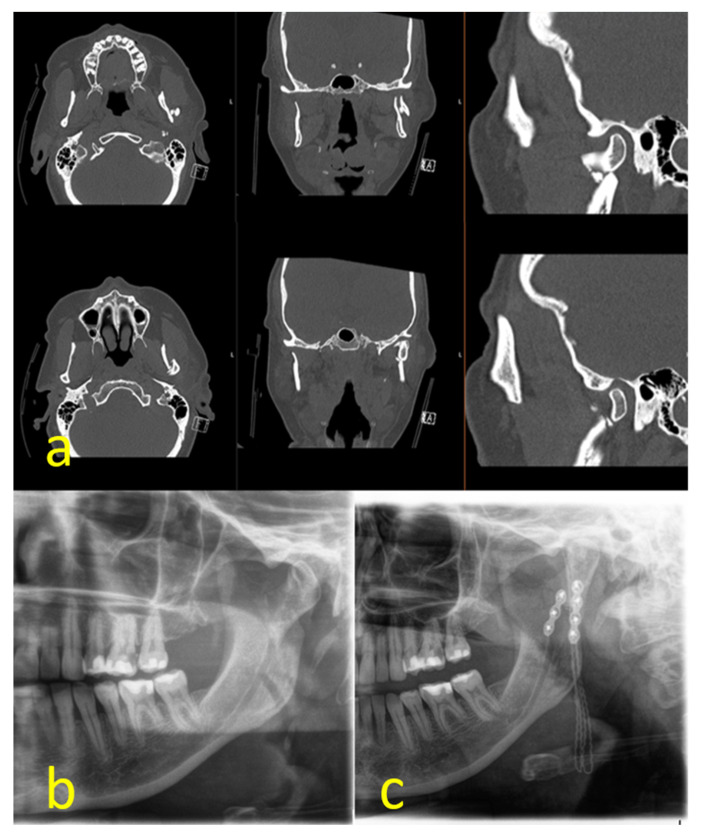
Comminuted type II fracture: CT-scan (**a**); preoperative panoramic x-ray (**b**); postoperative panoramic x-ray (**c**).

**Figure 3:**
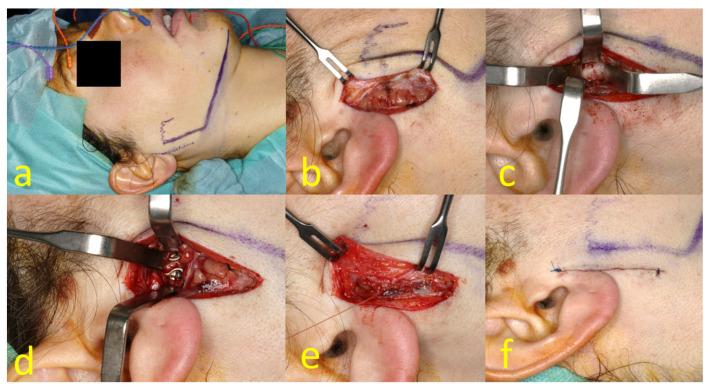
Retromandibular transparotid approach: preoperative skin marking, 4-channel neuromonitoring placed into the frontalis, orbicularis oculi and oris, and mentalis muscles (**a**); incision of parotid capsule (**b**); fracture reposition (**c**); ORIF in double-plating technique (**d**); watertight closure of parotid capsule (**e**); wound closure by intracutaneous suture (**f**).

The cases could be subdivided in 62 unilateral and 53 bilateral fractures. A total of 107 of the 115 fractures were treated by ORIF (93.0%); 8 underwent conservative therapy (7.0%). Conservative treatment by intermaxillary fixation was carried out for a mean of 23.57 ± 10.37 days. According to the Spiessl and Schroll classification, the dominant type of fracture was type II (*n* = 61; 53.0%) (Figure 1a) followed by type III (*n* = 22; 19.1%), type I and V (*n* = 15; 13.0% each), and type IV (*n* = 2; 1.7%). Type I was present in 62.5% of conservatively treated fractures (*n* = 5), whereas 97.0% of the displaced or dislocated fractures underwent ORIF (*n* = 97) (Figure 1b). Type IV and V fractures exclusively received open treatment. This distribution was statistically significant (*p* < 0.001). A total of 15 fractures were classified as comminuted fractures by reference to preoperative 3D imaging (Figure 2a–c). A retromandibular transparotid approach was chosen in 93.5% of the open-treated cases (*n* = 100) (Figure 3a–f).

### 3.2. Postsurgical Facial Nerve Palsy

In 11 of the 107 surgical cases, a postoperative paresis of the facial nerve was reported (10.3%); 7 patients (6.5%) showed temporary weakness of the facial nerve, which had a mean duration of 13.50 ± 13.41 weeks. A total of 4 of the patients showed a permanent paresis (3.7%). The most common appearance of a facial nerve paresis affected the zygomatic branch (54.5%; *n* = 6) followed by an affection of the temporal branch (27.3%; *n* = 3). Facial nerve palsy was significantly associated with female gender (*p* = 0.016), comminution fractures (*p* = 0.016), and high fractures (*p* = 0.021). Fracture classification (*p* = 0.142), displacement (*p* = 0.976), dislocation (*p* = 0.826), neuromonitoring (*p* = 0.062), approach (*p* = 0.596) and revision surgery (0.568) did not correlate with FNP (Table 3).

### 3.3. Post-Treatment Malocclusion

A significant increase in malocclusion was observed in bilateral fractures (41.5%) compared to unilateral fractures (22.6%) (*p* = 0.029), as well as in comminuted fractures (53.3%) (*p* = 0.048) 3 months after treatment. Mean postoperative subjective impairment in the case of post-treatment malocclusion was 4.36 *±* 2.50, rated by VAS. The occurrence of postoperative malocclusion was not significantly influenced by open or closed treatment (3 months: *p* = 0.695; 6 months: *p* = 0.546; 12 months: *p* = 0.751), fracture classification (*p* = 0.363; 0.844; 0.624), fracture displacement (*p* = 0.436; 0.554; 0.257), dislocation of the condylar head (*p* = 0.855; 0.317; 0.201), or fracture localization (*p* = 0.298; 0.723; 0.550). Concomitant mandibular fractures seem to be associated with the appearance of medium- and long-term post-treatment malocclusion (6 months: *p* = 0.029; 12 months: 0.012) (Table 4).

### 3.4. Post-Treatment Pain

A total of 53 patients (46.1%) did not report any pain 3 months after treatment, 44 patients (38.3%) complained pain in chewing and 18 patients (15.7%) reported durably occurring pain after surgical or non-surgical intervention. In the surgical group the mean duration of pain was 9.95 *±* 12.73 weeks versus 16.83 *±* 16.92 weeks in the conservative group. This difference in pain duration was not statistically significant (*p* = 0.22). Pain occurrence 3 months after treatment was also significantly lower in the open-treated group (*p* = 0.048). The overall pain intensity after treatment of extracapsular fractures of the mandibular condyle was reported to be 3.05 ± 2.35 at VAS. After surgery, the mean VAS level was 2.98 ± 2.42 versus 4.00 ± 1.07 after conservative treatment. This difference was statistically significant (*p* = 0.039). In total, 62 patients (53.9%) reported to have pain 3 months after treatment. In this period, post-treatment pain was significantly increased in females (69.6%; *p* = 0.006), in bilateral fractures (71.2%; *p* < 0.001) and in patients with further mandibular fractures (60.8%; 0.046). Thirty-three patients (28.7%) reported having pain 6 months after treatment. In this period, post-treatment pain was more frequent in polytraumatized patients (53.8%; *p* = 0.033) and in comminuted fractures (53.3%; *p* = 0.024) (Table 5). Twenty-two patients reported to have pain 12 months or more after treatment (19.1%). A significant association of pain lasting for 12 months or more with the assessed parameters could not be identified.

### 3.5. Post-Treatment Reduction in Maximum Mouth Opening

In 64 cases (55.6%), patients complained of reduced mouth opening after treatment for at mean 15.97 *±* 21.86 weeks. MMO reduction was significantly increased in female patients in all time periods (*p* < 0.001; *p* < 0.001; *p* = 0.011). A significant difference between conservative and surgical treatment could not be constituted.

In 33 patients, a reduction in mouth opening could be assessed after 3 months (28.7%), whereas in 24 patients MMO reduction was present after 12 months or more (20.8%). Three months after treatment, MMO reduction was significantly increased in bilateral fractures (*p* = 0.038). Six months after treatment, MMO reduction was significantly more frequent in bilateral fractures (*p* = 0.048), comminuted fractures (*p* = 0.024) and in patients with further mandibular fractures (*p* = 0.004). Comminution fractures were significantly associated with MMO reduction after 12 months (*p* = 0.001) (Table 6).

### 3.6. Further Discomfort

Sixteen patients (13.9%) reported suffering from cephalgia or pain in the neck muscles, respectively, and neck tension since the trauma, respectively, during treatment. This was significantly associated with long-term occlusal disharmony (*p* = 0.015), MMO reduction (*p* < 0.001), long-term pain at the fracture site (*p* < 0.001), and post-treatment mandibular deviation (*p* < 0.001). Thirteen patients reported a disturbing postoperative deviation of the mandible during opening movement. Ten of these thirteen patients (76.9%) also complained of long-term mouth opening reduction *p* < 0.001). Eight of them (61.5%) also suffered from post-treatment malocclusion (*p* < 0.001) and 9 of them (69.2%) reported long-term post-treatment pain (*p* < 0.001) (Table 7).

## 4. Discussion

Treatment of fractures of the mandibular condyle is discussed controversially. Among others FNP, malocclusion, hypomobility and persisting pain are the major complications possible after ORIF of condylar fractures [18,19,20] whereas pain, arthritis, open bite, deviation of the mandible on opening and closing movement, inadequate restoration of vertical height of the ramus leading to malocclusion, and ankylosis can go along with the closed treatment [21]. Nevertheless, some authors consider ORIF to be superior in subjective and objective functional clinical outcome in comparison to closed treatment and to be the treatment of choice at least for displaced fractures [21,22]. In the present study, we carried out a questionnaire survey including 115 patients presenting in our unit between 2006 and 2020 with uni or bilateral fractures of the mandibular condyle to gain information about post-treatment complications and discomfort. The study was carried out in the department of maxillofacial surgery at Regensburg University Hospital, Germany, which is a third-level hospital. Its facial trauma center has a commuting area of 2.2 million people in Eastern Bavaria. For this reason, we consider the study to be representative and purposive according to current standards. One hundred and seven fractures (93.0%) were treated by ORIF, whereas eight fractures (7.0%) received conservative therapy. This distribution of open and closed treatment is consistent with other studies [1]. Type one was present in 62.5% of conservatively treated fractures, whereas 97.0% of the displaced and dislocated fractures underwent ORIF (*p* < 0.001). It points out that surgical treatment is possible and appropriate in most cases, especially in cases of displaced or dislocated fractures.

In 31.3% of our cases, the patients reported a post-treatment malocclusion. Malocclusion seems to be a common subjective complication after treatment of extracapsular condylar fractures. Nevertheless, the number of occlusal disharmony is higher in our study compared to previous studies [23,24,25,26]. Our results point out that the subjective impression of occlusal disharmony may be stronger than the objective clinical assessment as performed in many studies. A significant difference between open and conservative treatment could not be displayed. However, it has to mentioned that comparison between surgery and conservative treatment is strongly limited by the low case number of the conservative group. Displaced and dislocated fractures were almost entirely treated by open approaches, whereas closed treatment focused mainly on not displaced fractures. Patients with bilateral condylar fractures seem to undergo a significantly higher risk to develop a malocclusion (*p* = 0.044). This finding is consistent with previous studies and might be caused by the different biomechanics and the more demanding reduction compared to unilateral fractures [4,27]. Comminuted fractures could also be identified as another predisposing factor for occlusal disharmony (*p* = 0.003). This might also be due to more challenging reposition and osteosynthesis.

The most concerning complication for an open reduction in condylar fractures is an injury to the facial nerve, which can cause temporary or permanent paralysis of the muscles of facial expression [28]. In our cohort, in total, 10.3% of the open-treated patients complained of a palsy of the facial. Permanent nerve damage occurred in 3.7% after ORIF. Rozeboom et al. reports an incidence of FNP of 12% with 95% recovery rate [11], whereas a meta-analysis by Al-Moraissi et al. identified the risk for permanent FNP after ORIF via retromandibular approach to be 1.4% [28]. Thean and Chang assessed 3.28% temporary and 0.82% permanent FNPs in a similar surgical cohort [24]. Our results suggest that subjective impression of facial nerve impairment also seems to be slightly higher than in objective clinical studies. The mean duration of temporary FNPs was 21.86 ± 25.27 weeks. The most frequently used approach was retromandibular transparotid which has also been identified to bear a certain risk for facial nerve damage. Nevertheless, the recovery rate of FNP after using the retromandibular transparotid approach is suggested to be higher compared to the anterior or posterior parotid approach. A possible explanation might be the necessity for less traction of the soft tissue and consecutively of the nerve [11]. According to other studies, we consider the risk to produce a permanent paresis of the facial nerve to be very low using the common approaches including the transparotid approach [11].

Comminution of the condyle seems to cause a higher incidence of post-surgical FNP maybe because of a time-consuming reposition and the need for wider approaches. In wider approaches, the risk for deliberate encountering branches of the facial increases. Al-Moraissi et al. identified encountering the facial nerve during surgical treatment of condylar fractures as a risk factor for FNP [29]. Other authors also identified traction of the nerve as a risk factor for postoperative palsy [11]. Interestingly, the zygomatic branch was affected the most often in cases of FNP. This correlates with the fact that high condylar fractures were associated with a higher risk for FNP (*p* = 0.021), as dissection in a more cranial direction has to be carried out. Al-Moraissi et al. also identified fractures located in condylar neck to carry a higher risk for FNP when treated via retromandibular approach [28]. Female gender is significantly more often associated with FNP (*p* = 0.016) compared to male gender. Imai et al. confirm this finding [30]. A possible explanation might be provided by an experimental model of neuronal regeneration, which indicates that testosterone accelerates functional recovery following FNP in male hamsters [31].

In our study, pain intensity after treatment was significantly reduced after ORIF compared to closed treatment (*p* = 0.039). This finding is in accordance with a meta-analysis conducted by Al-Moraissi and Ellis, which also displays lower VAS pain levels after the ORIF of condylar fractures [22]. Eckelt et al. also report significantly better treatment results in terms of less pain and discomfort for open treatment [32]. In our study, one month after treatment a significant higher percentage of patients reported pain in the conservative group (*p* = 0.048). Due to these findings, we suggest that ORIF is an effective way to decrease the level and duration of post-treatment pain, despite the type of fracture. Post-treatment pain was significantly increased in women for 0–3 months after therapy (*p* = 0.006). This finding may be due to the fact that female patients show a higher prevalence in general for temporomandibular joint pain, jaw muscle pain and neck muscle pain than male patients [33]. In 19.1% of the cases, the patients complained pain lasting for 12 months or longer after treatment. Chronic pain lasting longer than 12 months is reported to have a relevant neuropathic component [14]. In cases of chronification, pain was often expressed as cephalgia and pain or tension in the neck muscles. The connection between temporomandibular disorder and jaw injuries is well known [33,34]. Regarding the association between cephalgia and pain in the neck muscles in our study, with long-term occlusal disharmony we suggest that temporomandibular disorder might be induced or enforced by condylar mandibular fractures and their treatment. Occlusal disharmony often goes along with chronic orofacial pain [35]. Cephalgia and pain in the neck muscles may also be caused or increased by occlusal disharmony as a result of incorrect reposition in open or closed way of treatment. A symptom complex consisting of cephalgia or neck tension, occlusal disharmony, MMO restriction and mandibular deviation seems to be quite common after condylar fractures. Despite the incidence of long-term malocclusion being significantly higher in male patients (*p* = 0.018), the chronification of pain was similar in both genders in our study. Anyway, in comparison to other studies regarding pain after fractures in general [14], the incidence of chronic posttraumatic or postsurgical pain lasting for 12 months or longer after condylar fractures seems to be less than average.

Ellis identified mandibular hypomobility as a common complication after condylar fractures [4]. In our study, post-treatment mouth opening reduction occurred in 55.6% of our cases for 0–3 months, whereas a permanent reduction was reported by 20.9% of our patients, which made it the most common post-treatment complication. There was no significant difference detectable between open- and closed-treated patients. Ferretti et al. suggested that hematomas around the articular cavity of dislocated condylar fracture fragments, damage of the anatomic barrier, disc displacement, and long periods of limited mandibular mobility can induce ankylosis by vessel ossification [36]. In this context, comminution of the condyle could be identified as a risk factor for long-term MMO reduction (*p* = 0.001). Bone fragments, more demanding reposition and wider approaches may be responsible by potentially causing increased hematoma and a more extensive traumatization of soft tissue. Female patients stated to have a mouth opening decline significantly more frequently than male patients in short-, medium-, and long-term observation (*p* < 0.001; *p* < 0.001; *p* = 0.011). An obvious reason for this could not be identified. Maybe MMO reduction in females goes along with their higher post-treatment pain levels and their higher prevalence for chronic orofacial pain, respectively, temporomandibular disorder [33]. Bilateral fractures and further mandibular fractures seem to be associated with a significantly delayed recovery of MMO from 3–12 months (*p* = 0.048), maybe because of the significantly increased pain levels and both sided tissue traumatization they go along with.

In 11.3% of the cases, the patients reported to have a disturbing post-treatment deviation of the mandible during the mouth opening movement. This finding correlated significantly with long-term postoperative pain (*p* < 0.001), post-treatment malocclusion (*p* < 0.001) and long-term mouth-opening reduction (*p* < 0.001). Sarnat and Robinson stated that a subcondylar fracture in an adult may lead to lateral deviated mandible secondary to condylar remodeling from mechanical forces or condylar resorption from poor blood supply [37]. Deviation may also result from a one-sided posttraumatic joint malfunction and hypomobility, which leads to a movement to the fractured side. Gibstein et al. identified conservative treatment as a certain risk factor for mandibular deviation [2]. This finding could not be proved in our study.

One limitation of this study is the retrospective design based on a questionnaire survey. Another limitation of this study design is the poor response rate, as only 115 of 290 patients took part. This could lead to a relevant non-responder bias and to higher rates of complications compared to studies on the basis of objective clinical examination. Individual experience of the involved surgeons in open treatment of condylar fractures could not be assessed, despite the fact that it may have a big impact on the outcome. Treatment was carried out or supervised by a consultant-level surgeon in all cases. However, it could not be guaranteed that the same surgeon or surgical team performed the treatment of all the fractures mentioned above. A true comparison between conservative and surgical treatment is difficult regarding the small amount of closed treated fractures. Nearly two thirds of the condylar fractures were accompanied by further mandibular fractures. It is possible that post-treatment pain is caused by the concomitant fractures and not or not only by the condylar fracture. Another potential bias might be the high amount of bilateral fractures, with 46.1%. As bilateral fractures seem to cause more complications than unilateral fractures, it is possible that more patients with discomfort took part in the questionnaire survey than patients free of complaints.

## 5. Conclusions

MMO reduction is the most common subjective complication after treatment of extracapsular condylar fractures. The questionnaire evaluation led to a higher incidence of FNP and malocclusion, as perceived by the patients, than reported in studies based on objective assessment. This means that patients are subjectively more impaired by FNP and malocclusion than it appears in an objective medical examination. ORIF goes along with significantly lower pain levels and decreased pain duration. Surgical treatment seems to be appropriate for all types of extracapsular condylar fractures. Comminution of the condyle and bilateral fractures seem to be major risk factors for post-treatment complications. Female gender bears a higher risk for long-term MMO reduction, FNP and prolonged pain. A long-term symptom complex consisting of cephalgia or neck muscle tension, malocclusion, MMO reduction and mandibular deviation is possible after extracapsular condylar fractures.

## Figures and Tables

**Table 1:** Spiessl and Schroll classification of mandibular condylar fractures [15].

**Type I**	Fractures without displacement
**Type II**	Low fractures with displacement
**Type III**	High fractures with displacement
**Type IV**	Low fractures with dislocation
**Type V**	High fractures with dislocation
**Type VI**	Intracapsular fractures

**Table 2:** Baseline data.

Patients		115	
Sex			
Male		69 (60.0%)	
Female		46 (40.0%)	
Age		41.89 ± 16.59 years	
Polytrauma		13 (11.3%)	
Treatment			
Open		107 (93.0%)	
Closed		8 (7.0%)	
Spiessl and Schroll		Open treatment	Closed treatment
Type I	15 (13.0%)	10 (66.6%)	5 (33.3%)
Type II	61 (53.0%)	60 (98.4%)	1 (1.6%)
Type III	22 (19.1%)	20 (91.0%)	2 (9.1%)
Type IV	2 (1.7%)	2 (100%)	0 (0%)
Type V	15 (13.0%)	15 (100%)	0 (0%)
Unilateral		62 (53.9%)	
Bilateral		53 (46.1%)	
Concomitant mandibular fractures		74 (64.3%)	
Approach (in surgical cases)			
Retromandibular transparotid		100 (93.5%)	
Intraoral		3 (2.8%)	
Preexisting		1 (0.9%)	
Preauricular		1 (0.9%)	
Submandibular		1 (0.9%)	

**Table 3:** Postsurgical facial nerve palsy after ORIF.

Study Variable		Association with FNP (Ratio FNPs/Sample Size)		*p*-Value
**Total**		11/107 (10.3%)		-
**Temporary**		7/107 (6.5%)		
**Permanent**		4/107 (3.7%)		
**Sex**	Female	8/42 (19.0%)		0.016 *
	Male	3/66 (4.5%)		
**Fracture classification**	Type 1	1/10 (10%)		0.142
	Type 2	3/57 (5.2%)		
	Type 3	5/20 (25%)		
	Type 4	0/2 (0%)		
	Type 5	2/15 (13.3%)		
**Fracture displacement**	Yes	10/97 (10.3%)		0.976
	No	1/10 (10%)		
**Luxation of condylar head**	Yes	2/17 (11.8%)		0.826
	No	9/90 (10%)		
**Comminuted fracture**	Yes	4/14 (28.6%)		0.016 *
	No	7/93 (7.5%)		
**Fracture localization**	High	7/35 (20%)		0.021 *
	Low	4/72 (5.6%)		
**Neuromonitoring**	Yes	10/105 (9.5%)		0.062
	No	1/2 (50%)		
**Revision surgery**	Yes	0/3 (0%)		0.552
	No	11/104 (10.6%)		
**Approach**	Retromandibular transparotid	10/101 (9.9%)		0.596
	Other	1/6 (16.7%)		
**Localization of FNP**	Temporal branch	3/11 (27.3%)		-
	Zygomatic branch	6/11 (54.5%)		
	Buccal branch	2/11 (18.2%)		
**Association localization of FNP/localization of fracture**	High fractures	Temporal branch	2/35 (5.7%)	0.441
		Zygomatic branch	3/35 (8.6%)	
		Buccal branch	2/35 (5.7%)	
	Low fractures	Temporal branch	0/72 (0%)	
		Zygomatic branch	3/72 (4.2%)	
		Buccal branch	1/72 (1.4%)	

* significant at *p* < 0.05.

**Table 4:** Post-treatment malocclusion.

Study Variable		Post-Treatment Malocclusion (Ratio Malocclusion/Sample Size)			*p*-Value		
		3 Months	6 Months	12 Months	3 Months	6 Months	12 Months
**Total**		36/115 (31.3%)	24/115 (20.9%)	19/115 (16.5%)	-	-	-
**Sex**	Female	13/46 (28.3%)	6/46 (13.0%)	3/46 (6.5%)	0.566	0.092	0.018 *
	Male	23/69 (33.3%)	18/69 (26.1%)	16/69 (23.2%)			
**Treatment**	Open	33/107 (33.6%)	23/107 (21.5%)	18/107 (16.4%)	0.695	0.546	0.751
	Closed	3/8 (37.5%)	1/8 (12.5%)	1/8 (12.5%)			
**Unilateral fractures**		14/62 (22.6%)	11/62 (17.7%)	9/62 (14.5%)	0.029 *	0.372	0.531
**Bilateral fractures**		22/53 (41.5%)	13/53 (24.5%)	10/53 (18.9%)			
**Fracture classification**	Type 1	6/15 (40.0%)	4/15 (26.7%)	4/15 (26.7%)	0.363	0.844	0.624
	Type 2	15/61 (24.6%)	13/61 (21.3%)	10/61 (16.4%)			
	Type 3	10/22(45.5%)	5/22 (22.7%)	4/22 (18.2%)			
	Type 4	1/2 (50.0%)	0/2 (0%)	0/2 (0%)			
	Type 5	4/15 (26.7%)	2/15 (13.3%)	1/15 (6.7%)			
**Fracture displacement**	Yes	30/100 (30.0%)	20/100 (20.0%)	15/100 (15.0%)	0.436	0.554	0.257
	No	6/15 (40.0%)	4/15 (26.7%)	4/15 (26.7%)			
**Dislocation of condylar head**	Yes	5/17 (31.6%)	2/17 (11.8%)	1/17 (5.9%)	0.855	0.317	0.201
	No	31/98 (23.5%)	22/98 (22.4%)	18/98 (18.4%)			
**Comminuted fracture**	Yes	8/15 (53.3%)	4/15 (26.7%)	2/15 (13.3%)	0.048 *	0.554	0.721
	No	28/100 (28.0%)	20/100 (20.0%)	17/100 (17.0%)			
**Fracture localization**	High	14/37 (37.8%)	7/37 (18.9%)	5/37 (13.5%)	0.298	0.723	0.550
	Low	22/78 (28.2%)	17/78 (21.8%)	14/78 (17.9%)			
**Concomintant mandibular fractures**	Yes	25/74 (33.8%)	17/74 (23.0%)	17/74 (23.0%)	0.441	0.029 *	0.012 *
	No	11/41 (26.8%)	4/41 (9.8%)	2/41 (4.9%)			

* significant at *p* < 0.05.

**Table 5:** Post-treatment pain.

Study Variable		Association with Postoperative Pain (Ratio Postoperative Pain/Sample Size)			*p*-Value		
		3 Months	6 Months	12 Months	3 Months	6 Months	12 Months
**Total**		62/115 (53.9%)	33/115 (28.7%)	22/115 (19.1%)			
**Sex**	Female	32/46 (69.6%)	16/46 (34.8%)	9/46 (19.6%)	0.006 **	0.239	0.923
	Male	30/69 (43.5%)	17/69 (24.6%)	13/69 (18.8%)			
**Polytrauma**	Yes	10/13 (76.9%)	7/13 (53.8%)	5/13 (38.5%)	0.077	0.033 *	0.060
	No	52/102 (51.0%)	26/102 (25.5%)	17/102 (16.7%)			
**Treatment**	Open	7/8 (87.5%)	4/8 (50.0%)	3/8 (37.5%)	0.048 *	0.167	0.171
	Closed	55/107 (51.4%)	29/107 (27.1%)	19/107 (17.8%)			
**Unilateral fractures**		24/62 (38.7%)	18/62 (29.0%)	13/62 (21.0%)	<0.001 ***	0.931	0.588
**Bilateral fractures**		38/53 (71.7%)	15/53 (28.3%)	9/53 (17.0%)			
**Fracture classification**	Type I	9/15 (60.0%)	3/15 (20.0%)	2/15 (13.3%)	0.754	0.879	0.742
	Type II	32/61 (52.5%)	18/61 (29.5%)	13/61 (21.3%)			
	Type III	10/22 (45.5%)	6/22 (27.3%)	3/22 (13.6%)			
	Type IV	1/2 (50.0%)	1/2 (50.0%)	0/2 (0%)			
	Type V	10/15 (66.7%)	5/15 (33.3%)	4/15 (26.7%)			
**Fracture displacement**	Yes	53/100 (53.0%)	30/100 (30.0%)	20/100 (20.0%)	0.612	0.425	0.540
	No	9/15 (60.0%)	3/15 (20.0%)	2/15 (13.3%)			
**Dislocation of condylar head**	Yes	6/17 (35.3%)	6/17 (35.3%)	4/17 (23.5%)	0.334	0.515	0.617
	No	51/98 (52.0%)	27/98 (27.6%)	18/98 (18.4%)			
**Comminuted fracture**	Yes	11/15 (73.3%)	8/15 (53.3%)	5/15 (33.3%)	0.106	0.024 *	0.134
	No	51/100 (51.0%)	25/100 (25.0%)	17/100 (17.0%)			
**Revision surgery**	Yes	2/3 (66.7%)	1/3 (33.3%)	0/3 (0%)	0.653	0.857	0.393
	No	52/60 (86.7%)	32/112 (28.6%)	22/112 (19.6%)			
**Fracture localization**	High	20/37 (54.1%)	11/37 (29.7%)	7/37 (18.9%)	0.983	0.866	0.968
	Low	42/78 (53.8%)	22/78 (28.2%)	15/78 (19.2%)			
**Concomintant mandibular fractures**	Yes	45/74 (60.8%)	22/74 (29.7%)	16/74 (21.6%)	0.046 *	0.742	0.362
	No	17/41 (41.5%)	11/41 (26.8%)	6/41 (14.6%)			

* significant at *p* < 0.05, ** significant at *p* < 0.01 and *** significant at *p* < 0.001.

**Table 6:** Post-treatment MMO reduction.

Study Variable		MMO Reduction (Ratio MMO Reduction/Sample Size)			ukr		
		3 Months	6 Months	12 Months	3 Months	6 Months	12 Months
**Total**		64/115 (55.6%)	33/115 (28.7%)	24/115 (20.9%)	-	-	-
**Sex**	Female	35/46 (76.1%)	24/46 (52.2%)	15/46 (32.6%)	<0.001 ***	<0.001 ***	0.011 *
	Male	29/69 (42.0%)	9/69 (13.0%)	9/69 (13.0%)			
**Treatment**	Open	60/107 (56.1%)	30/107 (28.0%)	22/107 (20.5%)	0.739	0.568	0.766
	Closed	4/8 (50.0%)	3/8 (37.5%)	2/8 (25.0%)			
**Unilateral fractures**		29/62 (46.8%)	13/62 (21.0%)	13/62 (21.0%)	0.038 *	0.048 *	0.978
**Bilateral fractures**		35/53 (66.0%)	20/53 (37.7%)	11/53 (20.8%)			
**Fracture classification**	Type I	7/15 (46.7%)	3/15 (20.0%)	2/15 (13.3%)	0.612	0.695	0.745
	Type II	36/61 (59.0%)	16/61 (26.2%)	13/61 (21.3%)			
	Type III	10/22 (45.4%)	7/22 (31.8%)	4/22 (18.2%)			
	Type IV	1/2 (50.0%)	1/2 (50.0%)	1/2 (50.0%)			
	Type V	8/15 (53.3%)	6/15 (40.0%)	4/15 (26.7%)			
**Fracture displacement**	Yes	57/100 (57.0%)	30/100 (30.0%)	22/100 (22.0%)	0.453	0.425	0.441
	No	7/15 (46.7%)	3/15 (20.0%)	2/15 (13.3%)			
**Dislocation of condylar head**	Yes	10/17 (58.8%)	7/17 (41.2%)	5/17 (29.4%)	0.776	0.218	0.348
	No	54/98 (55.1%)	26/98 (26.5%)	19/98 (19.4%)			
**Comminuted fracture**	Yes	10/15 (66.7%)	8/15 (53.3%)	8/15 (53.3%)	0.357	0.024 *	0.001 **
	No	54/100 (54.0%)	25/100 (25.0%)	16/100 (16.0%)			
**Revision surgery**	Yes	1/3 (33.3%)	1/3 (33.3%)	1/3 (33.3%)	0.430	0.857	0.590
	No	63/112 (56.3%)	32/112 (28.6%)	23/112 (20.5%)			
**Fracture localization**	High	19/37(51.3%)	13/37 (35.1%)	8/37 21.6%)	0.523	0.228	0.891
	Low	45/78 (57.7%)	20/78 (25.6%)	16/78 (20.5%)			
**Concomintant mandibular fractures**	Yes	40/74 (54.1%)	28/74 (37.8%)	19/74 (25.7%)	0.643	0.004 **	0.088
	No	24/41 (58.5%)	5/41 (12.2%)	5/41 (12.2%)			

* significant at *p* < 0.05, ** significant at *p* < 0.01 and *** significant at *p* < 0.001.

**Table 7:** Further discomfort reported by patients.

		Cephalgia, Neck Tension		Mandibular Deviation	
			*p*-Value		*p*-Value
**Total**		16/115 (13.9%)	-	13/115 (11.3%)	-
**Malocclusion after 12 months**	Yes	6/19 (31.6%)	0.015 *	8/19 (42.1%)	<0.001 ***
	No	10/96 (10.4%)		5/96 (5.2%)	
**MMO reduction after 12 months**	Yes	7/24 (29.2%)	0.015 *	10/24 (41.7%)	<0.001 ***
	No	9/91 (9.9%)		3/91 (3.3%)	
**Pain after 12 months (in the area of fracture)**	Yes	9/22 (40.9%)	<0.001 ***	9/22 (40.9%)	<0.001 ***
	No	7/93 (7.5%)		4/93 (4.3%)	
**Chronic headache, neck tension**	Yes	-	-	6/16 (37.5%)	<0.001 ***
	No	-		7/99 (7.1%)	
**Mandibular deviation**	Yes	6/13 (46.2%)	<0.001 ***	-	-
	No	10/102 (9.8%)		-	

* significant at *p* < 0.05, *** significant at *p* < 0.001.

## Data Availability

Data can be obtained on request by the scientists who conducted the work independently from the industry. Data are not stored on publicly available servers.

## Supplementary Materials

The following supporting information can be downloaded at: https://www.mdpi.com/article/10.3390/jcm13051395/s1, Table S1: Questionnaire sent to patients.
