# Supplementary material for: Patient’s Perception of Outcome after Extracapsular Fractures of the Mandibular Condyle Differs from Objective Evaluation—Experience of a Third-Level Hospital
Source: J Clin Med. 2024 Feb 28;13(5):1395. doi: 10.3390/jcm13051395 (PMC10931758; doi:10.3390/jcm13051395)
Supplement: Supplementary file 1 [file jcm-13-01395-s001.zip › jcm-2850272-supplementary.pdf]

Universitätsklinikum Regensburg

Table S1

Questionnaire on the outcome after treatment of mandibular condylar fractures

Questionnaire no. …………

**Please describe the type of injury (e.g. fracture in the area of the right temporomandibular joint).**	…………………………………………………………………………………………………
**Please enter your age at the time of the trauma.**	…………………………………………………………………………………………………
**Please enter your gender.**	…………………………………………………………………………………………………

Please check all that apply.

**Type of fracture treatment:**		
Were the jaws "strapped"? (e.g. screws in the jaw and rubber bands or wire)	Yes ☐	No ☐
Was surgery performed under general anesthesia?	Yes ☐	No ☐
Did a second operation have to be performed to improve the result?	Yes ☐	No ☐

**Pain after treatment**				
Is there or was there pain in the area of the fracture/surgical site/temporomandibular joint after the treatment (surgery, strapping, ...)?			Yes ☐	No ☐
How long did you feel pain after the treatment?		☐ I felt pain after 3 months.	☐ I felt pain after 6 months.	☐ I felt pain after 12 months.
When does or did the pain occur?	When chewing ☐	Always ☐	There is or was no pain. ☐	

How severe is or was your pain?										
10 (Strongest pain imaginable)	9	8	7	6	5	4	3	2	1	0 (No pain)
☐	☐	☐	☐	☐	☐	☐	☐	☐	☐	☐

**How well do or did your upper and lower teeth fit together when you bit down after the treatment?**			
It is the same as before the jaw fracture.	Yes ☐		No ☐
The teeth do or did not fit together as they did before the fracture.	Yes ☐		No ☐
Approximately how long did the teeth not fit together after the treatment?	☐ My teeth did not fit after 3 months.	☐ My teeth did not fit after 6 months.	☐ My teeth did not fit after 12 months.

If your teeth no longer fit together, how severely are or were you affected?

Universitätsklinikum Regensburg

10 (Most severe possible impairment)	9	8	7	6	5	4	3	2	1	0 (No impairment)
☐	☐	☐	☐	☐	☐	☐	☐	☐	☐	☐

**Is or was there any impairment of the facial muscles after the treatment?**		
It is or was the same as before the jaw fracture.	Yes ☐	No ☐
There is or was mobility disorder in the forehead.	Yes ☐	No ☐
There is or was a disturbance when closing the eye.	Yes ☐	No ☐
There is or was a mobility disorder when moving the mouth (especially the upper lips) or puffing cheeks.	Yes ☐	No ☐
There is or was a mobility disorder when moving the mouth (especially the lower lips and the corner of the mouth) or do you have an asymmetrical smile?	Yes ☐	No ☐

Approximately how long has the impairment of your facial movement lasted?	☐ I felt an impairment after 3 months	☐ I felt an impairment after 6 months	☐ I felt an impairment after 12 months

**Do you have problems opening your mouth?**			
It is or was same as before the jaw fracture.	Yes ☐		No ☐
The mouth does or did not open as far as normal after the operation.	Yes ☐		No ☐
Approximately how long did the problems with opening your mouth last?	☐ I had problems to open my mouth after 3 months.	☐ I had problems to open my mouth after 6 months.	☐ I had problems to open my mouth after 12 months

**Additions (e.g. further discomfort, problems, abnormalities, ...)**
....
....
....

Thank you very much for your help!
